# G-Quadruplex Binders Induce Immunogenic Cell Death Markers in Aggressive Breast Cancer Cells

**DOI:** 10.3390/cancers11111797

**Published:** 2019-11-15

**Authors:** Sarah Di Somma, Jussara Amato, Nunzia Iaccarino, Bruno Pagano, Antonio Randazzo, Giuseppe Portella, Anna Maria Malfitano

**Affiliations:** 1 Department of Translational Medical Sciences, University of Naples Federico II, 80131 Naples, Italy; sarah.ds@libero.it; 2 Department of Pharmacy, University of Naples Federico II, 80131 Naples, Italy; jussara.amato@unina.it (J.A.); nunzia.iaccarino@unina.it (N.I.); bruno.pagano@unina.it (B.P.); antranda@unina.it (A.R.)

**Keywords:** G-quadruplex, breast cancer, immunogenic cell death markers, T cell activation

## Abstract

Background: DNA G-quadruplex (G4) structures represent potential anti-cancer targets. In this study, we compared the effect of two G4-targeting compounds, C066-3108 and the gold standard BRACO-19. Methods: In breast and prostate cancer cells, cytotoxicity induced by both molecules was measured by a sulforhodamine B assay. In breast cancer cells, cycle, apoptosis, the formation of G4 structures, calreticulin and high mobility group box 1 (HMGB1), as well as T cell activation, were analyzed by flow cytometry and adenosine triphosphate (ATP) by luminescence. Results: Both ligands inhibited cell survival and induced DNA damage. In MCF-7 cells, G4 ligands increased the subG0/G1 phase of the cell cycle inducing apoptosis and reduced intracellular ATP. In untreated MCF-7 cells, we observed a slight presence of G4 structures associated with the G2/M phase. In MDA-MB231 cells, G4 ligands decreased the G1 and enhanced the G2/M phase. We observed a decrease of intracellular ATP, calreticulin cell surface exposure and an increase of HMGB1, accompanied by T cell activation. Both compounds induced G4 structure formation in the subG0/G1 phase. Conclusions: Our data report similar effects for both compounds and the first evidence that G4 ligands induce the release of danger signals associated with immunogenic cell death and induction of T cell activation.

## 1. Introduction

In the human genome, polymorphic guanine (G)-rich sequences can fold into G-quadruplex (G4) secondary structures, characterized by the formation of the so-called G-tetrads, planar cyclic arrays of four guanine bases linked by Hoogsteen hydrogen bonds [1]. Two or more G-tetrads can stack on top of each other to form a right-handed quadruple-helical structure that in recent years attracted a lot of attention as an anti-cancer target. G4 structures are not randomly distributed in the human genome, but are detected at the end of chromosomes, the telomeres, and also cluster in promoter regions of relevant proto-oncogenes, such as *c-MYC* [2], *BCL-2* [3], *KRAS* [4] and *c-KIT* [5], likely regulating oncogene expression. In particular, the formation of G4 structures at telomeres prevents telomerase access to the G-rich single strand, thus inhibiting telomeres extension. Moreover, stabilization of G4 structures with specific ligands induced DNA damage at telomeres along with the induction of cancer cell senescence and apoptosis [6]. Targeting G4 structures by means of selective small molecules is a key challenge to elicit a therapeutic response and the focus of clinical investigation. To this aim, several classes of ligands able to bind and stabilize G4 structures have been described so far [7,8,9].

**Figure 1:**
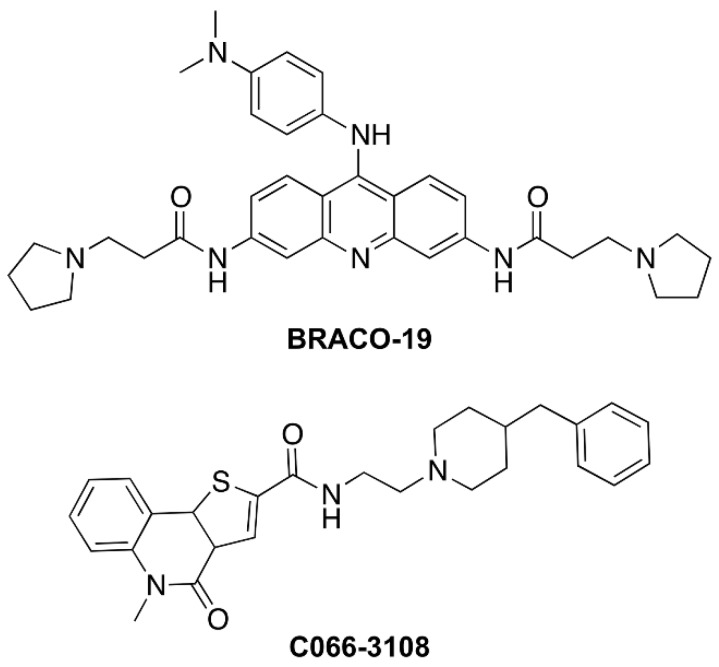
Chemical Structure of C066-3108 and BRACO-19. The chemical structure of C066-3108 and BRACO-19 is reported.

2,6-pyridine-dicarboxamide (PDCA) derivatives showed induction of apoptosis and alteration of the cell cycle in glioma cell lines, effects related to telomere instability [10]. Pyridostatin was found to be able to induce DNA damage, reduce the levels of the proto-oncogene tyrosine–protein kinase *SRC* and the SRC-dependent motility of breast cancer cells, thus promoting the arrest of cell growth and of the cell cycle in human cancer cells [11]. Berberine derivatives arrested both the cell growth and cycle along with senescence induction and DNA damage at the telomere region in cancer cells [12]. Some carbazole derivatives showed a significantly higher cytotoxicity in breast cancer cells than in non-tumorigenic breast epithelial cells, although this effect was not associated with telomerase inhibition [13]. Recently, G4 stabilization in the promoter region of some oncogenes by benzimidazole-carbazole ligands was suggested to reduce cancer risk through the loss of function of proteins coded by these genes. Indeed, these compounds repressed oncogene expression and displayed cell-specific cytotoxicity in Hela and MCF-7 cancer cells [14]. Among G4 ligands that have entered clinical trials, there are CX-5461 and CX-3552. The former, CX-5461, a multiple G4-stabilizer with a specific toxicity against BRCA1/2 deficient tumors, is currently in advanced phase I clinical trials [15]. CX-3552, more commonly known as quarfloxin, is a ribosomal-G4 targeting compound that inhibits rRNA biogenesis by preventing G4 interaction with nucleolin. Actually, quarfloxin is the only G4 ligand that has reached Phase II clinical trials, but it was withdrawn due to bioavailability-related problems [16]. BRACO-19 and C066-3108 (Figure 1) are two other examples of G4-targeting ligands with high affinity and good selectivity toward telomeric G4. BRACO-19, a 3,6,9-trisubstituted acridine derivative, is a well characterized potent and selective ligand of telomeric G4 with the ability to inhibit telomerase activity and exert antitumor effects [17,18,19]. In fact, BRACO-19 inhibited cell growth and induced senescence in 21NT breast cancer cells along with the reduction of telomerase activity, and also exerted an in vivo anti-tumor effect when administered to mice bearing a vulval carcinoma [19]. Induction of extensive DNA damage response at telomeres and senescence by BRACO-19 have been observed also in human glioblastoma cells [20]. On the other hand, C066-3108 is an interesting bioactive G4 ligand discovered by some of us in 2013. Its 5,9b-dihydrothieno[3,2-*c*]quinolin-4(3a*H*)-one scaffold displayed an impressive telomeric G4 binding and stabilizing activity, with no detectable binding to G4-forming sequences at other genomic sites. This behavior correlates with its ability to induce selective DNA damage at telomeres, reduce cell growth and trigger apoptosis in cancer cells [21].

The release of DNA damage response signals like damage-associated molecular patterns (DAMPS), differently by apoptosis, is also involved in the initiation of the immunogenic cell death (ICD) and stimulation of an anti-tumor immune response [22,23,24,25], however the induction of this cell death mechanism has never been associated with G4 structures.

In this context, we focused on two telomeric-G4 targeting compounds, C066-3108 and BRACO-19. We addressed their effects in breast cancer, because this type of tumor is the most lethal in women. In particular, the triple negative breast cancer, lacking of the estrogen receptor (ER), progesterone receptor (PR) and human epidermal growth factor receptor 2 (HER2), is resistant to therapies, and is associated with reduced survival for tumor recurrence and metastasis [26]. Thus, we compared the effects of C066-3108 and BRACO-19, investigating potential different effects in less (hormone-dependent) and high aggressive (hormone-independent) breast cancer cells, MCF-7 and MDA-MB231, respectively. The investigation of their anti-cancer activity has been explored, highlighting different cell death mechanisms; in particular we focused on the induction of ICD along with both C066-3108’s and BRACO-19’s ability to promote/stabilize G4 structure formation in the nuclei. This field of investigation fills an area of G4 binding small molecules that is unexplored, and provides evidence of potential induction of immunogenicity against aggressive breast cancer.

## 2. Results

### 2.1. Anti-Proliferative Effects of G4 Ligands in Cancer Cells

**Figure 2:**
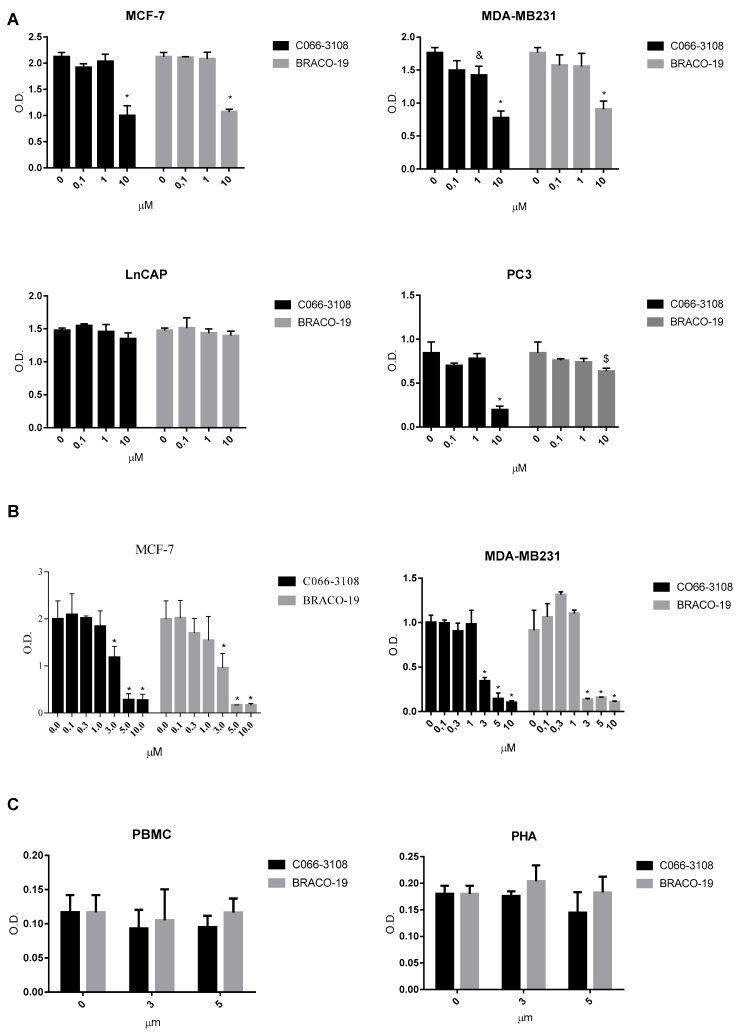
Cytotoxic effects of G4 ligands. (**A**) Cytotoxic effect of G4 ligands determined by SRB assays in MCF-7, MDA-MB231, PC3 and LNCaP cell lines at 72 h of treatment with C066-3108 and BRACO-19 and (**B**) in MCF-7 and MDA-MB231 cells at six days of treatment. Figures report cell viability (mean of three independent experiments as optical density, O.D.) generated with different concentrations of G4 ligands. The statistical significance was calculated by GraphPad Prism 7 with two-way Analysis of Variance (ANOVA) using Dunnett’s multiple comparisons test (* *p* < 0.0001, ^&^ *p* < 0.005, ^$^ *p* < 0.01). (**C**) Survival determined by MTT assay of resting and/or PHA-activated PBMC cultured in the presence and in the absence of G4 ligands at the concentrations of 3 and 5 µM for 5 days. Figures report O.D. indicative of cell survival (data are the mean of three independent experiments). No statistical difference was observed with respect to the untreated control as calculated by GraphPad Prism 7.

In order to compare the anti-proliferative effects of C066-3108 and BRACO-19 in several cancer cell lines, we selected prostate and breast cancer cells. Initially, to ascertain more responsive cell lines we used less aggressive LNCaP and MCF-7 cells and highly aggressive PC3 and MDA-MB231 cells. Cells were cultured for 72 h in the presence and in the absence of G4 ligands, and we observed cytotoxicity in PC3, MCF-7 and MDA-MB231 cells at the highest ligand concentration, whereas no effect was detected in LNCaP (Figure 2A). This initial screening helped us to select breast cancer cells for further experiments. We extended the time of culture and increased the concentration of the ligands. In particular, we cultured MCF-7 and MDA-MB231 cells for six days after G4 ligand treatment at concentrations ranging from 0.1 to 10 µM. We observed a dose-dependent inhibition of cell survival at low micromolar concentrations by SRB assay. Similar anti-proliferative effects ranging from 3 to 10 µM were observed in both cell lines (Figure 2B). Thus, we used 3 and 5 µM ligand concentrations to investigate other effects. To ascertain that these G4 ligands did not exert unspecific cytotoxic effect or induced cytotoxicity in normal human cells, we used peripheral blood mononuclear cells (PBMC) isolated from healthy donors. Cell survival was assessed after six days of culture by MTT assay. We observed that both resting and/or phytohemagglutinin (PHA)-activated PBMC were not affected by compounds treatment (Figure 2C), thus highlighting that these molecules are not toxic in healthy human primary cells. Indeed, we showed that G4 ligands used at 3 µM were not cytotoxic also in the normal breast cell line MCF-10A (Figure S1).

### 2.2. C066-3108 and BRACO-19 Induce DNA Damage in Breast Cancer Cells

**Figure 3:**
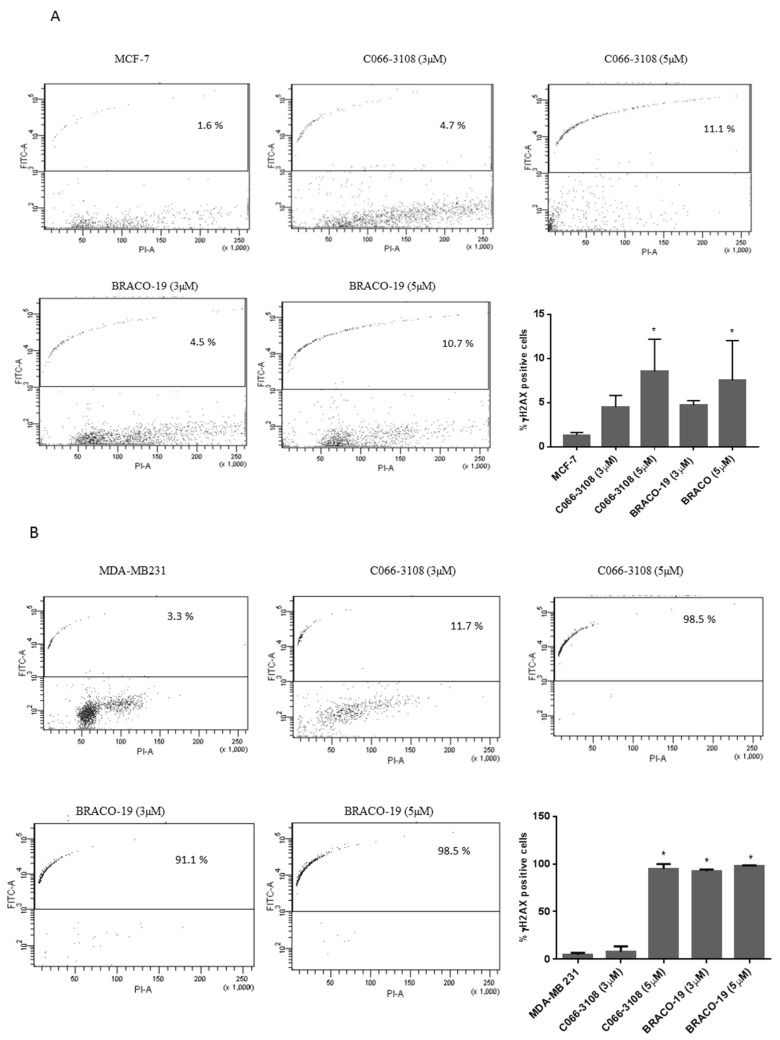
G4 ligands induce DNA damage in breast cancer cells. MCF-7 (**A**) and MDA-MB231 (**B**) cells were treated with G4 ligands at the indicated concentrations. The dot plot profiles indicate in the upper fluorescein isothiocyanate (FITC) positive panel the amount of DNA damage indicated by yH2AX staining. On our *x* axis the PI positivity is reported to analyze specific DNA staining. The dot plots reported are representative of a single experiment, whereas the histograms represent the mean ± standard deviation (SD) of at least three independent experiments. The statistical significance was calculated by GraphPad Prism 7 with one-way ANOVA with Dunnett’s multiple comparisons test ((**A**) * *p* < 0.005, (**B**) * *p* < 0.0001).

In other cell systems, C066-3108 and BRACO-19 have been reported to induce DNA damage at telomeres [20,21]. Herein, we planned to better define their effects on cell death, analyzing the induction of DNA damage on both MCF-7 and MDA-MB231 cell lines. At this purpose, we performed a kinetic analysis at three and six days after treatment. After three days no DNA damage was observed (Figure S2), whereas after six days, we observed that in MCF-7 cells, γH2AX positivity was relatively low at the highest concentrations of both G4 ligands (C066-3108 ~8.5% of cells and BRACO-19 ~7.5% of cells, Figure 3A). Conversely, in MDA-MB231 higher levels of DNA damage were observed with C066-3108 (at 5 µM ~94.9% of cells) and BRACO-19 (at 3 µM ~92.5% of cells and at 5 µM ~98.2% of cells, Figure 3B).

### 2.3. C066-3108 and BRACO-19 Arrest Cell Cycle Progression

**Figure 4:**
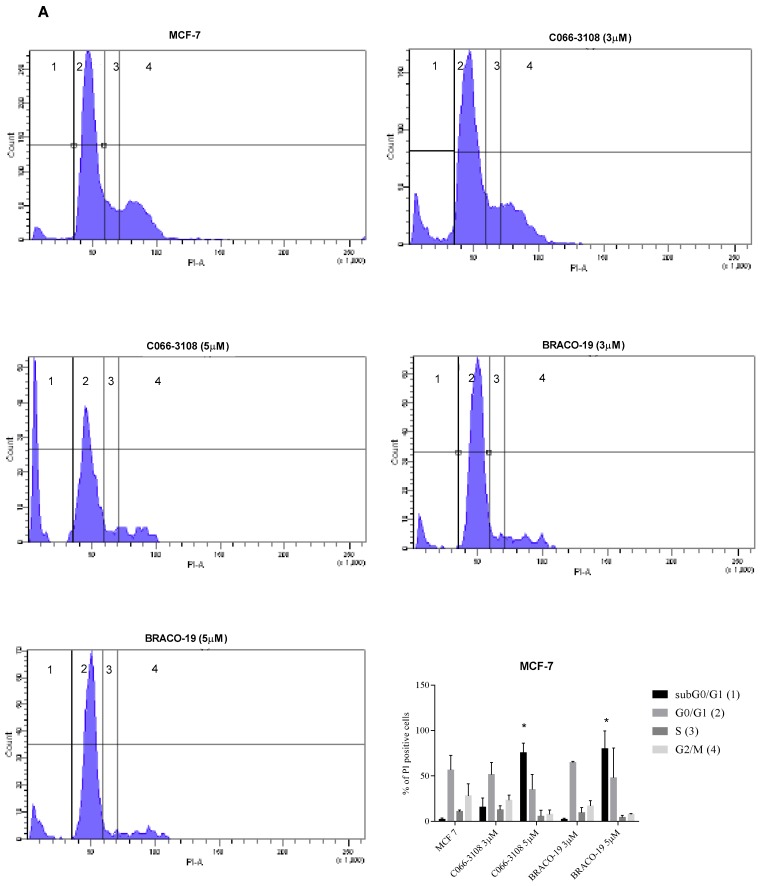

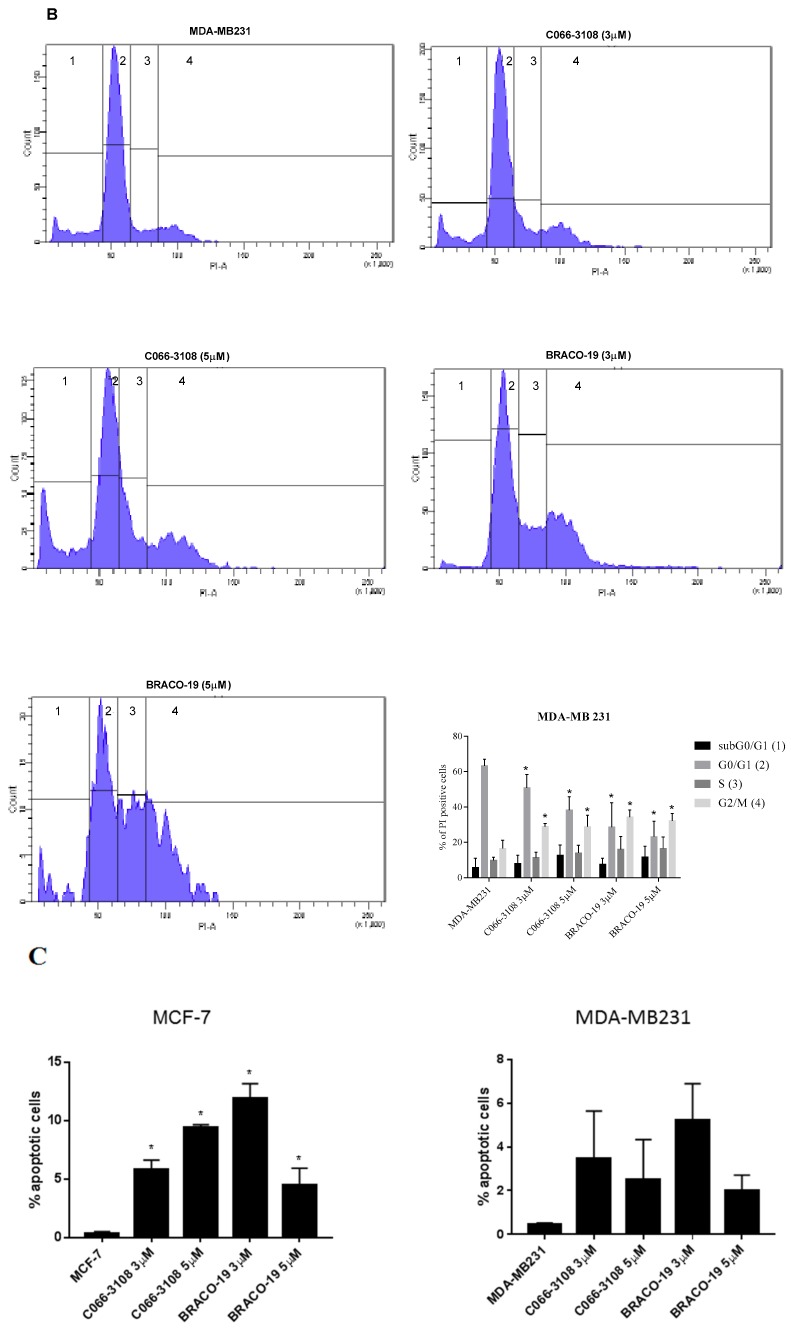
Inhibition of cell cycle progression and apoptosis induction by G4 ligands. MCF-7 (**A**) and MDA-MB231 (**B**) cells were treated with G4 ligands at the indicated concentrations. Cells were stained with PI to evaluate cell cycle progression. A representative flow cytometry profile of cell cycle is shown for both cell lines, and the percent of cells in each phase of the cell cycle is indicated for the single experiment, whereas the bars in the histograms represent the mean ± SD of at least three independent experiments. The statistical significance was calculated by GraphPad Prism 7 with two-way ANOVA using Sidak’s multiple comparisons test ((**A**) * *p* < 0.0001, (**B**) * *p* < 0.05). Apoptosis induction (**C**) was evaluated by annexin V/PI staining. The percent of early apoptotic cells is reported in the histograms representing the mean ± SD of five independent experiments. The statistical significance was calculated by GraphPad Prism 7 with one-way ANOVA with Dunnett’s multiple comparisons test (* *p* < 0.005).

To better characterize the effects of both molecules on cell cycle progression, we evaluated a kinetic of treatment at three and six days of culture. After three days of treatment, we detected no difference with respect to untreated control cells (Figure S3A,B). Conversely, different effects on the cell cycle between MCF-7 (Figure 4A) and MDA-MB231 (Figure 4B) cells were observed after six days of treatment. In particular, in MCF-7 cells, C066-3108 and BRACO-19 at the highest concentration increased the subG0/G1 phase, indicative of cell death. On the other hand, in MDA-MB231 cells both compounds decreased the G0/G1 phase and enhanced the G2/M phase. We used N(6)-isopentenyladenosine (IPA) as our control compound, previously shown to be able to increase the subG0/G1 and the G2/M phases and reduce the G0/G1 phase of the cell cycle in MDA-MD231 cells [27] (Figure S3C).

### 2.4. Apoptosis Induction in MCF-7 Cells by Flow Cytometry

In order to investigate the mechanisms of cell death, we addressed whether G4 ligands induced apoptosis in breast cancer cells. We performed in both cell lines a kinetic at three and six days after treatment with G4 ligands. In MCF-7 and in MDA-MB231 cells, at three days of culture we did not observe difference with respect to the control (Figure S4A). Of note, at six days of treatment we detected apoptosis induction at 3 and 5 µM ligand concentration in MCF-7 cells, in particular observing an increase of early apoptotic cells. Conversely, in MDA-MB231 cells, no effect was detected (Figure 4C). As control of apoptosis induction in MCF-7 cells, we used IPA at the concentration of 5 µM (Figure S4B).

### 2.5. Increase of Calreticulin Surface Exposure in MDA-MB-231 Cells

**Figure 5:**
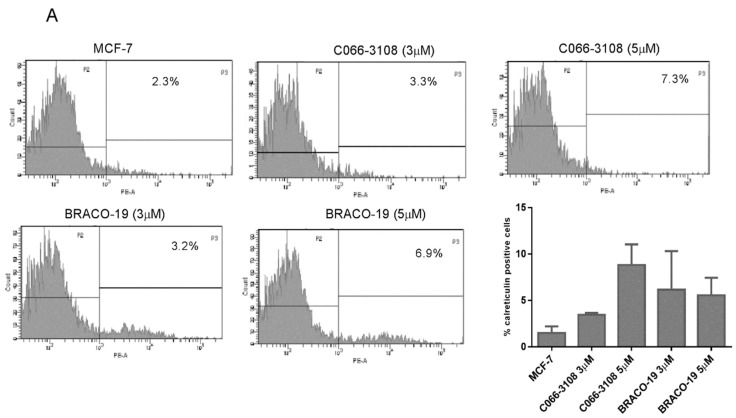

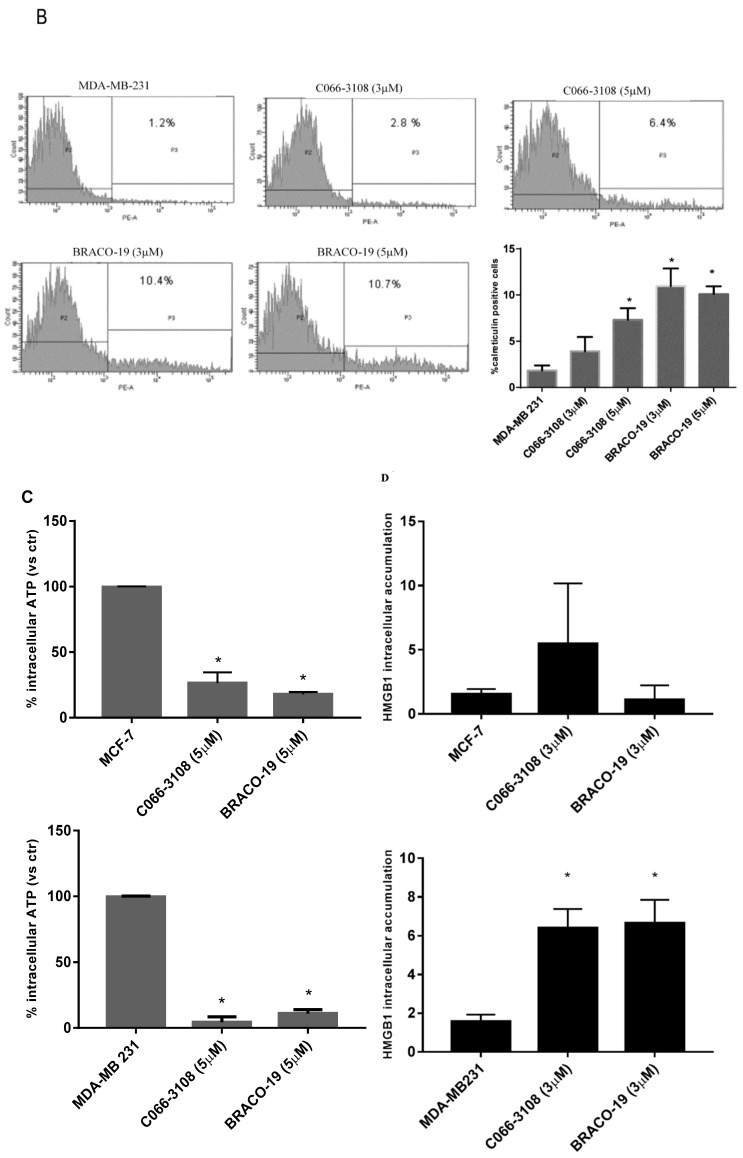
ICD induction by G4 ligands. ICD hallmarks were evaluated in MCF-7 and MDA-MB 231 cells treated with G4 ligands. Calreticulin expression as cell surface marker was evaluated in MCF7 (**A**) and MDA-MB231 (**B**) cells. Representative flow cytometry profiles of a single experiment are reported, whereas the histograms represent the mean ± SD of three independent experiments (* *p* < 0.005). ATP intracellular release by MCF-7 and MDA-MB231 treated with G4 ligands at 5 µM was determined as luminescence by microwell plate reader (**C**) the histograms represent the mean ± SD of three independent experiments (* *p* < 0.05). HMGB1 intracellular accumulation is reported in the histograms for MCF-7 and MDA-MB231 cells at the concentration of 3 µM (histograms report the mean ± SD of three independent experiments, *p* < 0.05) (**D**). The statistical significance was calculated by GraphPad Prism 7 one-way ANOVA with Dunnett’s multiple comparisons test.

To investigate other cell death mechanisms potentially induced by the herein investigated G4 ligands, we assessed the induction of ICD able to prime anti-tumor immune response and characterized by three specific hallmarks, calreticulin surface exposure, ATP and high mobility group box 1 (HMGB1) release. At this purpose, we analyzed the expression of calreticulin in MCF-7 and MDA-MB231 cells at three and six days after treatment. No difference was detected with respect to untreated control cells after three days of treatment (Figure S5A). Results obtained after six days of treatment with G4 ligands are reported in Figure 5. Both C066-3108 and BRACO-19 did not induce significant calreticulin exposure in MCF-7 (Figure 5A). Conversely, a slight but significant induction of cell-surface translocation of calreticulin at 5 µM of C066-3108 and both 3 and 5 µM of BRACO-19 (Figure 5B) was observed in MDA-MB231. 

### 2.6. C066-3108 and BRACO-19 Decrease Intracellular ATP Release

Extracellular ATP release generated from dying cancer cells represents a strong “find-me” signal to recruit immune cells. Thus, we evaluated changes of ATP level analyzing its intracellular decrease after 48 h (Figure S5B) and six days (Figure 5C) of treatment. 

In both cell lines, C066-3108 and BRACO-19 at the concentration of 5 µM time-dependently, reduced intracellular ATP, which was evaluated as luminescence signal.

### 2.7. C066-3108 and BRACO-19 Effects on Intracellular HMGB1

Finally, we investigated the release of HMGB1 after three and six days of G4 ligand treatment. Noteworthy, HMGB1 was recently demonstrated to bind G4 DNA structures [28,29] thus we analyzed its intracellular accumulation. After 72 h no difference was observed with respect to untreated cells (Figure S5C), whereas after six days, the accumulation of HMGB1 was significantly detected in the presence of C066-3108 (3 µM) and BRACO-19 (3 µM) only in MDA-MB231 cells (Figure 5D).

### 2.8. BRACO-19 Induces T-Cell Activation

**Figure 6:**
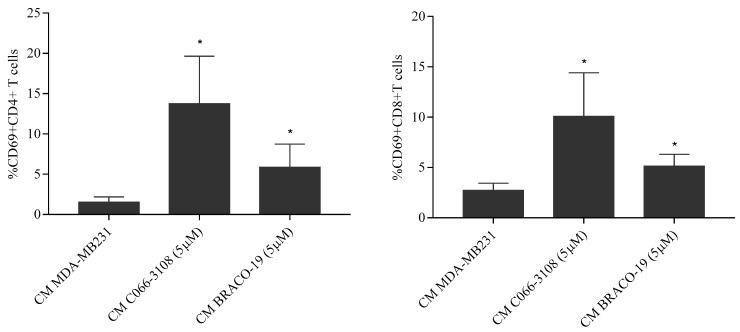
T cell activation by G4 ligands. CM obtained from the experimental conditions indicated in the figure were used to evaluate T cell activation induced by G4 ligands. The figure reports CD69+CD4+T cells and CD69+CD8+T cells. The histograms represent the mean ± SD of five independent experiments. The statistical significance was calculated by GraphPad Prism 7 with one-way ANOVA with Turkey’s multiple comparisons test (* *p* < 0.05).

In order to address the efficacy of ICD marker induction in terms of immune cell activation, we evaluated CD69 activation marker in human CD4+ and CD8+ T cells. Diluted (1:1) conditioned medium (CM) of C066-3108 and BRACO-19 at the concentration of 5 µM was used to stimulate the PBMC of female healthy donors for 48 h. The dilution was necessary to maintain at least half of the volume of RPMI that is the PBMC optimal growing media. We observed activation of both CD8 and CD4+T cells. Data of five responsive donors are reported in Figure 6.

### 2.9. G4 Structure Induction/Stabilization in Breast Cancer Cells

**Figure 7:**
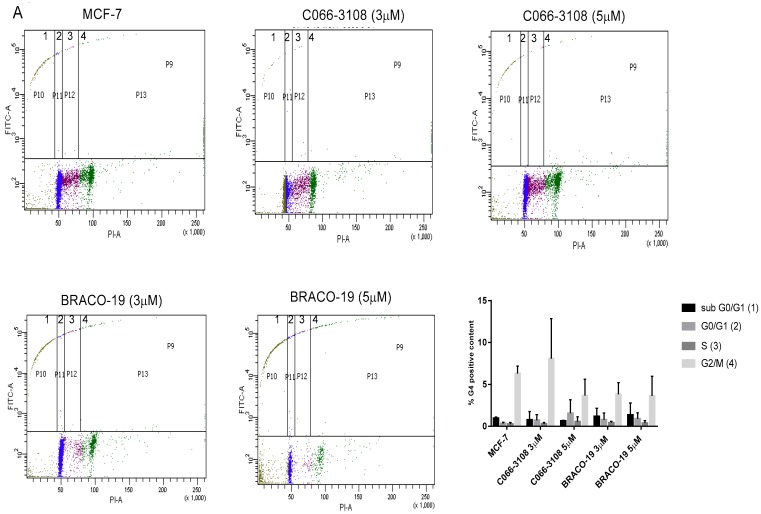

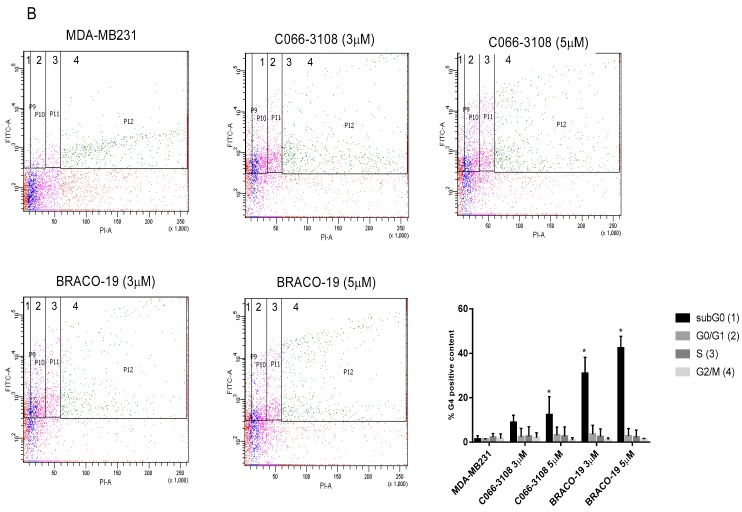
G4 ligand stabilization of G4 structures. G4 ligand stabilization in MCF-7 (**A**) and MDA-MB231 (**B**) cells was evaluated by flow cytometry detecting by gating strategies in each phase of the cell cycle on PI positive cells (PI-W versus PI-H dot plot) the percent of G4 structure positivity. Flow cytometry profiles representative of a single experiment are reported, whereas the histograms represent the mean ± SD of five independent experiments. The statistical significance was calculated by GraphPad Prism 7 with two-way ANOVA with Dunnett’s multiple comparisons test (* *p* < 0.05).

In order to address whether the effects observed with G4 ligands might be correlated with G4 structure stabilization and/or induction, we examined the content of G4 structures in the DNA of both cell lines in the presence and absence of the ligands. The presence of G4 structures was examined by flow cytometry gating on the different phases of the cell cycle (dot plot PI-W vs. PI-H) to associate to each phase the amount of G4 structures (dot plot FITC vs. PI). In basal conditions, G4 structures were detectable only in the G2/M phase of MCF-7 cells. In MDA-MB231 cells, the treatment with both the compounds enhanced the amount of G4 structures in the subG0/G1 phase of the cell cycle. The effect was significant at the highest concentration of C066-3108 and at both the concentrations of BRACO-19 (Figure 7).

## 3. Discussion

The formation of dynamic G4 structures in the human genome prompted the development of novel, small molecules able to selectively interact and stabilize these motifs to elicit an anti-tumor response. Numerous G4 ligands have been developed so far and tested to assess their affinity, binding potential and ability to reduce cancer cell growth by DNA damage and cell death. Up to date, apoptosis induction is the main mechanism reported for G4 ligands, however, it is known that DNA damage response is also involved in eliciting ICD [22,23,24,25], a mechanism not previously investigated in the case of G4 ligands. Numerous studies also suggest the anti-cancer potential of G4-targeting molecules. Nevertheless, because of their poor selectivity profile and drug-like properties, none of them advanced through the drug discovery pipeline. In this study, we used breast cancer cells that previously showed to be a responsive cell target of G4 ligands [13,14,19]. In particular, BRACO-19 and C066-3108 (Figure 1) were selected as they represent interesting examples of telomeric-targeting G4 ligands capable of stabilizing such structures with different binding modes. Indeed, BRACO-19 binds to the external G-tetrads by π-stacking interaction [30], while our previous studies indicate that C066-3108 has a mixed binding mode (i.e., external-stacking and groove binding) [21]. To get a comparison with other less and highly aggressive cell lines, we used LNCap and PC3 cells of prostate cancer. Both compounds exhibited a cytotoxic effect in MCF-7, MDA-MB231 and PC3 cell lines at the highest concentration, whereas no effect was detected in LNCaP cells (Figure 2A). Thus, we selected breast cancer cells and extended the time of culture and concentrations. BRACO-19 and C066-3108 induced a dose response cytotoxic effect in MCF-7 and MDA-MB231 after six days of incubation (Figure 2B). In order to check tumor cell specificity, we tested potential inhibitory growth effects in freshly isolated PBMC from healthy donors. We observed no inhibition of cell viability in resting and/or PHA-activated PBMC (Figure 2C), suggesting no toxic activity in healthy primary cells and also in the normal breast cell line MCF-10A (Figure S1). Furthermore, the induction of DNA damage was detected by γH2AX intracellular staining. In both cell lines at 72 h of culture, no effect was observed with respect to untreated control cells. After six days of treatment, in MCF-7 cells, both the compounds induced little DNA damage that was significant at the highest concentration used (Figure 3A). At variance, in MDA-MB231 cells, both compounds strongly induced DNA damage (Figure 3B). These data are in agreement with previous findings providing evidence of the ability of G4 ligands to induce DNA damage in cancer cells [11,12,20]. The different effects observed in MCF-7 and MDA-MB231 cells suggest that the latter cells are not able to elicit a DNA damage response to induce repair mechanisms and are more sensitive to G4 ligands. Likely, these small molecules work efficiently in more invasive cancer cell lines, as shown in both breast and prostate cancer cells. This result is particularly interesting because it provides evidence that targeting G4 structures is an efficient approach in highly aggressive and metastatic cells like MDA-MB231 cells. To better characterize the cytotoxic effects of both drugs, we analyzed the cell cycle progression. As expected, we observed the different behavior of G4 ligands in the two cell lines after six days of treatment, whereas 72 h treatment did not affect the cell cycle in both cell lines. In particular, in MCF-7 cells we detected a significant increase of the subG0/G1 phase at the highest concentration of C066-3108 and BRACO-19, indicating their ability to induce cell death. In MDA-MB231 cells, both compounds reduced the cell population in the G1 phase along with accumulation in the G2/M phase; however, the subG0/G1 phase was only slightly but not significantly increased (Figure 4A,B). To understand the apparent discrepancy between the two cell lines, such as higher DNA damage and lack of significant increase of the subG0/G1 phase in MDA-MB231 cells and lower DNA damage and increase of subG0/G1 phase in MCF-7 cells, we investigated the induction of apoptosis. AnnexinV/PI staining was performed three days after treatment, and according to the results obtained from the cell cycle assays, no effects were detected.

After six days, only in MCF-7 cells we could observe a significant increase of early apoptotic cells, whereas no effect was reported in MDA-MB231 cells (Figure 4C). In agreement with the effect observed in MCF-7 cells, a previous study showed that C066-3108 was able to increase the subG0/G1 phase of the cell cycle and induce apoptosis in HT29 colorectal adenocarcinoma cells [21]. In order to investigate the different mechanism of action of the two G4 ligands in MDA-MB231 cells, we evaluated ICD, a kind of cell death that primes immune cells against cancer cells. Three specific molecules appear to be required for optimal immune priming against malignant cells: the membrane localization of calreticulin, and the release of HMBG1 and ATP into the tumor microenvironment [31]. Thus, we assessed the induction of calreticulin as cell surface marker and observed that in MCF-7 cells it was not exposed (Figure 5A). Conversely, in MDA-MB231 cells calreticulin surface translocation was induced at 5 µM C066-3108 and both at 3 and 5 µM BRACO-19 after six days of treatment (Figure 5B). Another hallmark representative of ICD is the release of ATP that rapidly declines when cells die, due to either necrosis or apoptosis. Thus, we analyzed ATP intracellular content following treatment with C066-3108 and BRACO-19, and observed a significant time-dependent decrease of ATP, both in MCF-7 and MDA-MB231 cell lines. The effect was similar between C066-3108 and BRACO-19 in MCF-7 cells, whereas in MDA-MB231 the reduction was slightly higher with C066-3108 (Figure 5C). The third hallmark of ICD is HMGB1. It predominantly accumulates in nuclei and binds chromatin in all cells; however, it can shuttle between the nucleus and cytoplasm, and when its nuclear localization signal is modified, it migrates in the cytosol, cell membrane and extracellular space [32]. Recently, HMGB1 was demonstrated to be a novel G4 interactor able to recognize these DNA structures at chromosome ends with good specificity and without unfolding them [33]. In the same study, HMGB1 protein was observed to colocalize in the cell nucleus with the telomere repeat binding factor 1 (TRF1) and with an anti-G4 antibody, recognizing for more than 80%, the telomeres [33]. In addition, HMGB1 was also shown to be able to bind and stabilize the *KRAS* promoter G4, suggesting its potential role in the oncogene transcriptional regulation via the functional recognition of G4 structures [28]. Thus, we analyzed HMGB1 intracellular accumulation, whose presence might suggest that it binds stabilized or induced G4 structures in the nuclei. In MCF-7 cells, HMGB1 was not produced, and the G4 ligands did not change its levels with respect to untreated cells, whereas in MDA-MB231 cells, both compounds induced intracellular HMGB1 accumulation (Figure 5D). Our data provide the first evidence that G4 ligands are able to regulate all main signals required for ICD induction in MDA-MB231 cells. To ascertain that ICD markers were efficient in eliciting an immune response, we evaluated a CD69 activation marker in CD4+ and CD8+ T cells. In five donors, we observed the induction of T-cell activation in the presence of C066-3108 and BRACO-19 (Figure 6), thus suggesting that it might be able to elicit an immune response although HMGB1 might not be released in the extracellular phase at this stage. In future studies it will be helpful to extend the number of donors and kinetic of response and evaluate the phenotype of antigen presenting cells. Finally, in order to correlate the effects observed with G4 stabilization, we assessed whether our molecules affected the content of G4 structures in cells. Both the cell lines showed low basal levels of G4 structures slightly more pronounced in the G2/M phase of the cell cycle in MCF-7 cells. No significant effect on G4 structure induction was observed in MCF-7 cells upon the addition of C066-3108 and BRACO-19. Conversely, in MDA-MB231 cells, C066-3108 induced G4 structures at both concentrations with a significant effect at the highest concentration; however, the induction of G4 structures was more pronounced with BRACO-19 at all the concentrations used. Both the compounds determined the induction of G4 structures in the subG0/G1 phase of the cell cycle, suggesting that enhanced G4 DNA content is found in dying cells (Figure 6), and accordingly both compounds increased nuclear HMGB1. Our data suggest a more specific G4-mediated effect in MDA-MB231 cells associated with the induction of ICD markers. It might be speculated that accumulation of HMGB1 in the nuclei might be ineffective for immune cell recruitment, however, we suggest that calreticulin surface translocation and ATP release might be sufficient to recall immune cells and prime their anti-cancer response. Nonetheless, we cannot exclude that HMGB1 might be later passively released; further studies to assess this issue are ongoing in our laboratory. 

Notably, ICD induction is a mechanism elicited by other chemotherapetuic agents, in particular platinum-based drugs are known to bind a wide array of molecules including DNA, and several findings support the assumption that DNA is the most important platinum target. Among these compounds oxaliplatin induces full ICD, whereas cisplatin induces ATP and HMGB1 release; thus it is commonly combined with radiotherapy to compensate for the lack of calreticulin exposure [34]. The relevance of G4 structures as anticancer target is also supported by the evidence that currently used chemotherapeutic drugs, like the anthracyclines daunomycin and doxorubicin, have been shown to bind both DNA quadruplex and duplex in solution [35]. Indeed it is emerging that these agents are also involved in interaction with telomeric DNA quadruplex that drives to senescence and cell death in cancer cells [36]. These findings further support the relevance of studies aimed to investigate the therapeutic efficacy of new and highly selective G4 binders.

## 4. Materials and Methods

### 4.1. Cells and Drugs

Prostate (LNCaP and PC3) and breast (MCF-7 and MDA-MB231) cancer cell lines were cultured in DMEM (GIBCO, Paisley, UK). Growth media were supplemented with 2 mM L-glutamine, 50 ng/mL, streptomycin, 50 units/mL penicillin, and 10% heat-inactivated fetal bovine serum (FBS) (GIBCO). MCF-10A were cultured in DMEM-1640 10% FBS supplemented with 2 mM L-glutamine, 50 ng/mL, streptomycin, 50 units/mL penicillin, epithelial growth factor (EGF) 40 ng/mL and insulin 5 µg/mL. Cells were maintained in a humidified atmosphere with 5% CO_2_ at 37 °C. Cells were harvested with 0.25% trypsin (Sigma-Aldrich, St Louis, MO, USA) at 70–80% of confluence. Cells undergoing exponential growth were used for all the experiments. PBMC from female healthy donors were separated over Lymphoprep gradients (Lonza, Walkersville, MD, USA). PBMC were grown in RPMI 1640 (GIBCO), supplemented with 2 mM L-glutamine, 50 ng/mL, streptomycin, 50 units/mL penicillin, and 10% heat-inactivated FBS. C066-3108 and BRACO-19, were dissolved in dimethyl sulfoxide (DMSO) and stored at −20°. IPA (kindly provided by Dr. C. Laezza) was dissolved in DMSO.

### 4.2. Sulforhodamine B (SRB) and MTT Proliferation Assays

SRB assay was performed with MCF-7, MDA-MB231, LNCAP, PC3 and MCF-10A cell lines. MCF-7 and MDA cells were seeded in triplicates in 96-well plates at a density of 2000 cells/well while LNCAP and PC3 at density of 5000 cells/well to perform assays at 72 h. MCF-7 and MDA-MB231 at a density of 500 cells/well and MCF-10A at a density of 1000 cells/well were used for the assays at six days. Cells were allowed to adhere for 24 h. After the incubation, G4 ligands were added to the culture at concentrations ranging from 0.1 to 10 µM. At the end of the treatment, cells were fixed with 50% *v*/*v* trichloroacetic acid for two hours under stirring at 4 °C. After incubation, cells were washed with distillated water. Plates were dried overnight and stained with 0.4% *w*/*v* SRB in 1% *v*/*v* acetic acid at room temperature for 30 min on shaker. Washes were made with 1% acetic acid, until the removal of the unbound dye and the plates were left to dry. The dye was solubilized with TRIS-HCl 10 mM. The reading was performed at 495 nm by spectrophotometer as previously described [37].

For MTT assays PBMC were cultured in triplicates in 96-well plates (1 × 10^5^ cells × well) in the presence and absence of 1.5% PHA (Sigma-Aldrich). C066-3108 and BRACO-19 were added at the concentrations of 3 and 5 µM for five days. After the treatment, 10 µL per well of MTT solution were added in the last 3 or 4 h of incubation. Stop solution was added to each well and absorbance was read within 1 h at 570 nm according to the Cell growth determination kit MTT based (Sigma) and as previously described [38].

### 4.3. γH2AX and/or G4 and PI Intracellular Staining

MCF-7 and MDA-MB231 cell lines were cultured in 24-well plates (8000 cells) in the presence and absence of C066-3108 and BRACO-19 at the concentrations of 3 and 5 µM for six days. After the incubation, cells were detached with trypsin, washed twice with PBS, fixed in 70% (*v*/*v*) ethanol and stored at −20 °C at least overnight. 

The cell pellet was washed with PBS/Tween buffer (PBT) (0.5% *w*/*v* BSA and 0.1% *v*/*v* Tween 20 in PBS) and re-suspended in PBT containing γH2AX antibody (Millipore, #05-636, 1:100, Billerica, MA, USA) and or anti-G4 1H6 antibody (Millipore, MABE 1126, 1:100). After 1 h incubation at room temperature, samples were washed with PBT and re-suspended in PBT containing Alexa Fluor 488 anti-mouse (Invitrogen, USA, #A11001, 1:100) at darkness. After further 30 min, cells were washed with PBT (0.5% BSA, 0.1% Tween 20 in PBS) and re-suspended in propidium iodide (PI, Sigma) (0.015 mol/L) for 20 min. Flow cytometry acquisition was performed for the emission in FL1 and FL3 channels with a BD LSRFortessa (BD Biosciences, San Jose, CA, USA) and analyses were obtained using BD FACSDiva Software (bdbiosciences.com).

### 4.4. Cell cycle, Annexin V/PI and Calreticulin Staining

MCF-7 and MDA-MB231 cells were cultured as indicated above with G4 ligands for three and six days. After the incubation with G4 ligands and IPA as control compound, cells were detached with trypsin or PBS/EDTA for Annexin V staining and washed with PBS. For cell cycle, cells were fixed in 70% (*v*/*v*) ethanol at least overnight. Pellets were washed with PBS and re-suspended in PBS containing RNaseA (Roche) (0.4 U) and PI (0.015 mol/L), incubated for 20 min at room temperature and analyzed for emission in the FL3 channel. To remove artifacts, doublets and aggregates, an electronic doublet discrimination was performed using the area versus width of the fluorescence (FL3) pulse. For apoptosis induction, collected cells were washed with Annexin V binding buffer (Biolegend, San Diego, CA, USA). The pellet was re-suspended in 50 μL of Annexin V binding buffer containing fluorescein isothiocyanate (FITC)-conjugated Annexin V (1:40 Biolegend, #640906). After 15 min of incubation at room temperature, 250 μL PI solution (0.0015 mol/L in PBS) was added to each sample just before analysis. For calreticulin staining, cells were collected washed with PBS and stained with anti-calreticulin-PE conjugated (1:100, Enzo Life Sciences, New York, NY, USA) for 15 min on ice. After the incubation, cells were washed with PBS and analyzed by flow cytometry.

### 4.5. ATP Intracellular Concentration

MCF-7 and MDA-MB231 cells (2000 cells × well) were cultured in duplicates in 96-well plates. After 24 h of incubation, C066-3108 and BRACO-19 were added to the cells at the concentration of 5 µM for further 48 h. After the incubation, mammalian cell lysis solution which opens up the cells, allowing the intracellular adenosine triphosphate (ATP) to be released, was added. After 5 min of shaking, substrate solution was added, plates were shaken for 5 min and finally dark adapted for 10 min before measuring luminescence. This assay evaluates light emission caused by the reaction of ATP with luciferase and D-luciferin. The emitted light is proportional to the concentration of ATP (ATPlite, Perkin Elmer, MA, USA).

### 4.6. HMGB1 Intracellular Staining

MCF-7 and MDA-MB231 cells (2000 cells × well) were left to adhere for 24 h. G4 ligands at the concentration of 3 µM were added to the culture for three and six days. After the incubation, cells were detached with trypsin, washed with PBS, fixed 10 min at room temperature in PFA 10%, washed in PBS, permeabilized in methanol 90%, then transferred to ice 10 min, and kept at −20 °C before intracellular staining. Cells were then washed and re-suspended in FACS buffer (BSA 0.2%, NaN_3_ 0.05% in PBS), incubated with HMGB1-PE antibody for 30 min at room temperature, washed in PBS, re-suspended in FACS buffer and analyzed by flow cytometry.

### 4.7. T-cell Activation Assays

PBMC (1 × 10^6^ cells × mL) isolated from five healthy female donors were cultured for 48 h with the CM (1:1 in RPMI 10%FBS) of MDA-MB231 cells in the presence and in the absence of C066-3108 (5 µM) and BRACO-19 (5 µM). After the incubation, cells were collected and stained with CD4FITC, CD8APC and CD69PE (Myltenyi, Bergisch Gladbach, Germany). Cells were incubated on ice for 15 min, then washed and re-suspended in PBS for flow cytometry acquisition.

## 5. Conclusions

Overall, our data suggest a similar action of both compounds. In addition, we report the first evidence that G4-binding small molecules induce specifically in the aggressive MDA-MB231 cell line ICD markers associated with induction of T-cell activation and G4 structure formation. The data reported in this study (summarized in Table 1) may open a new area of future investigation addressing the immunological relevance in G4 structure induction and suggest G4 ligands as good candidates against breast cancer.

## Figures and Tables

**Table 1:** C066-3108 and BRACO-19 activity in breast cancer cell lines.

G4 Ligand Effects	MCF-7	MDA-MB231
Anti-proliferative effects	√	√
yH2AX phosphorylation	√	√
Apoptosis	√	x
Immunogenic cell death markers: Reduction of ATP Calreticulin exposure Enhanced intracellular HMGB1	√ x x	√ √ √
T-cell activation	not investigated	√
G4 structure induction	x	√

## Supplementary Materials

The following are available online at https://www.mdpi.com/2072-6694/11/11/1797/s1, Figure S1: Cytotoxicity in MCF-10A, Figure S2: DNA damage in MCF-7 and MDA-MB231, Figure S3: Cell cycle progression in MCF-7 and MDA-MB231, Figure S4: Apoptosis in MCF-7 and MDA-MB231, Figure S5: ICD hallmarks in MCF-7 and MDA-MB 231 cells treated with G4 ligands.

## Abbreviations

G-quadruplex	G4
High mobility group box 1	HMGB1
Immunogenic cell death	ICD
Estrogen receptor	ER
Progesterone receptor	PR
Human epidermal growth factor receptor 2	HER2
Damage-associated molecular patterns	DAMPS
Peripheral blood mononuclear cells	PBMC
Sulforhodamine B	SRB
Phytohemagglutinin	PHA
Conditioned media	CM
Propidium iodide	PI
Telomere repeat binding factor 1	TRF1
