# Supplementary material for: G-Quadruplex Binders Induce Immunogenic Cell Death Markers in Aggressive Breast Cancer Cells
Source: Cancers (Basel). 2019 Nov 15;11(11):1797. doi: 10.3390/cancers11111797 (PMC6895816; doi:10.3390/cancers11111797)
Supplement: Supplementary file 1 [file cancers-11-01797-s001.pdf]

Supplementary Materials

G-Quadruplex Binders Induce Immunogenic Cell Death Markers in Aggressive Breast Cancer Cells

Sarah Di Somma, Jussara Amato, Nunzia Iaccarino, Bruno Pagano, Antonio Randazzo, Giuseppe Portella and Anna Maria Malfitano

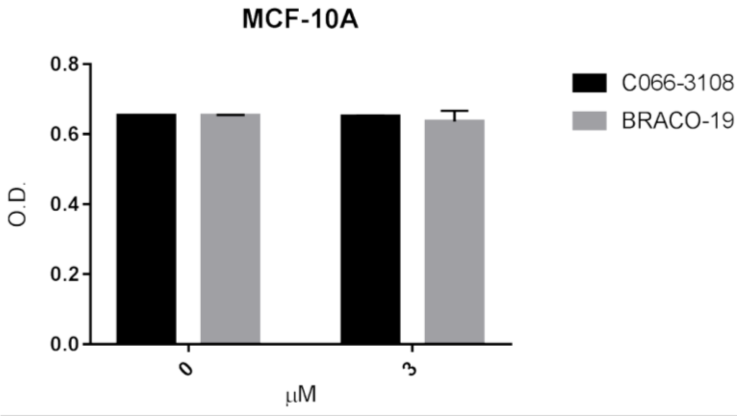

Figure S1. Cytotoxicity in MCF-10A. Cytotoxic effect of C066-3108 and BRACO-19 determined by SRB assays in MCF-10A at 6 days of treatment. Figure reports cell viability (as optical density, O.D.) generated with 3 µM of G4 ligands. No statistical difference (calculated by GraphPad Prism 7 with two-way Anova using Sidak's multiple comparisons) was observed with respect to the untreated control.

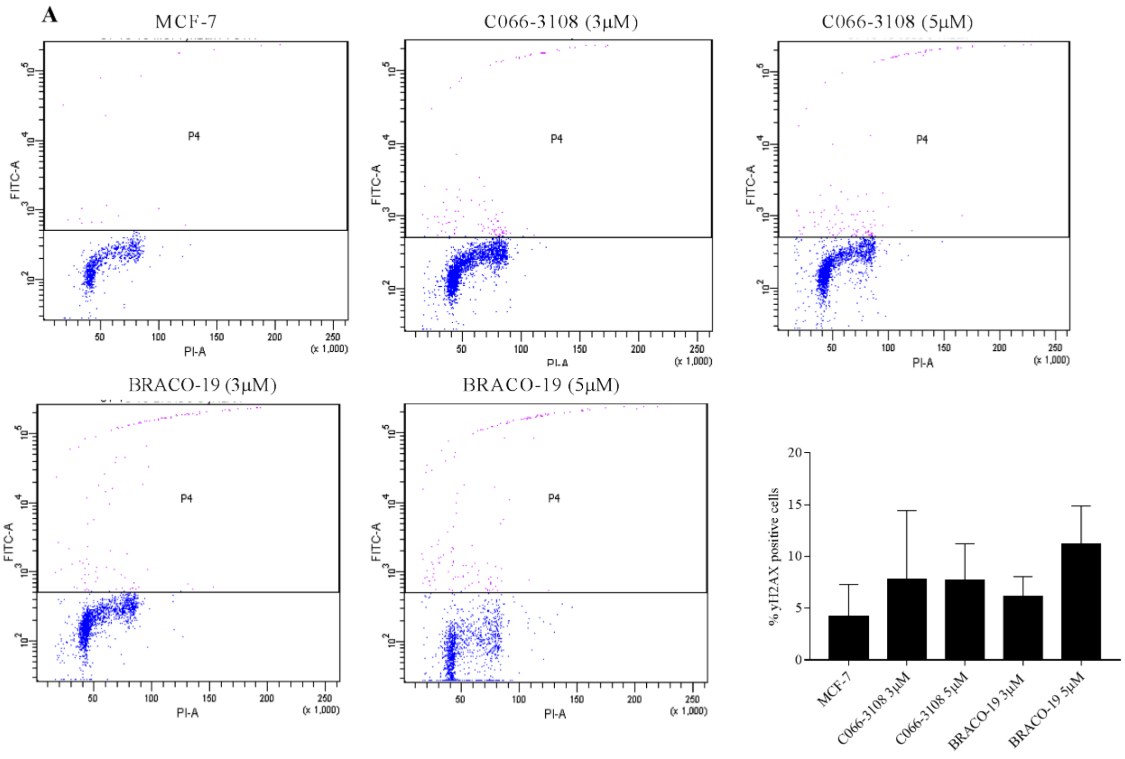

B

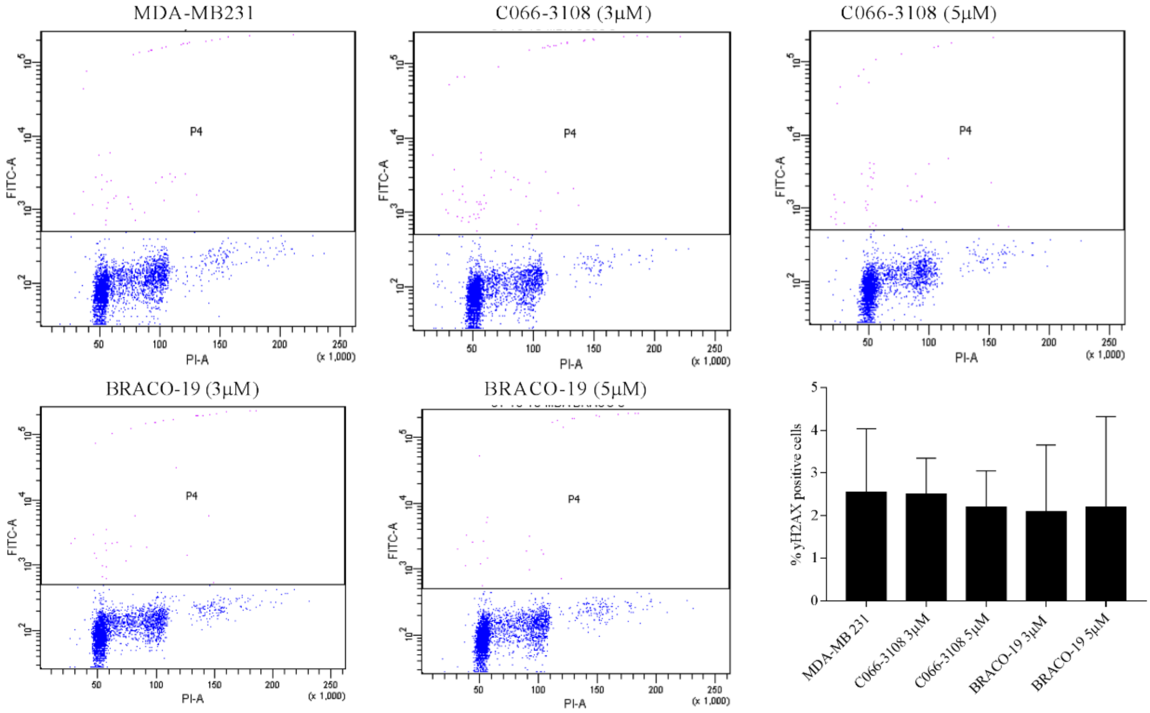

Figure S2. DNA damage in MCF-7 and MDA-MB231. MCF-7 (**A**) and MDA-MB231 (**B**) cells were treated with G4 ligands at the indicated concentrations. The dot plot profiles indicate in the upper FITC positive panel the amount of DNA damage indicated by γH2AX staining. On x axis PI positivity is reported to analyze specific DNA staining. The dot plots reported are representative of a single experiment whereas the histograms represent the mean ± st.dev. of at least three independent experiments. No statistical significance was detected (calculated by GraphPad Prism 7 with one-way Anova with Dunnett's multiple comparisons test).

A

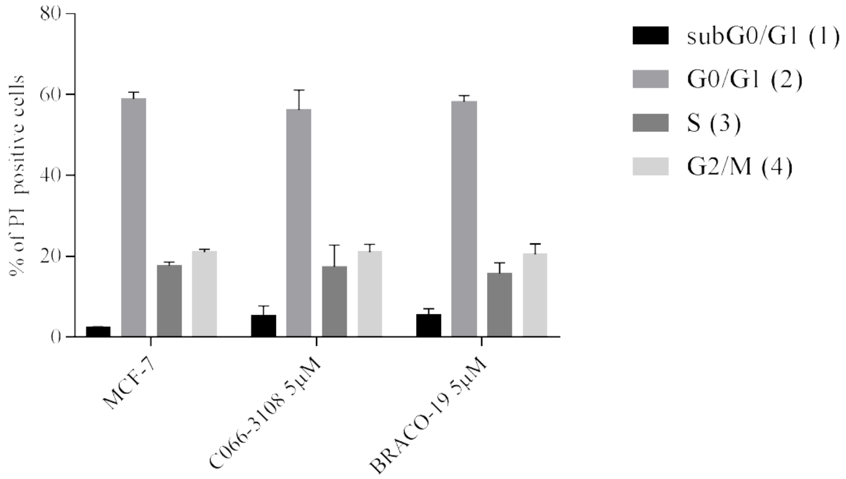

B

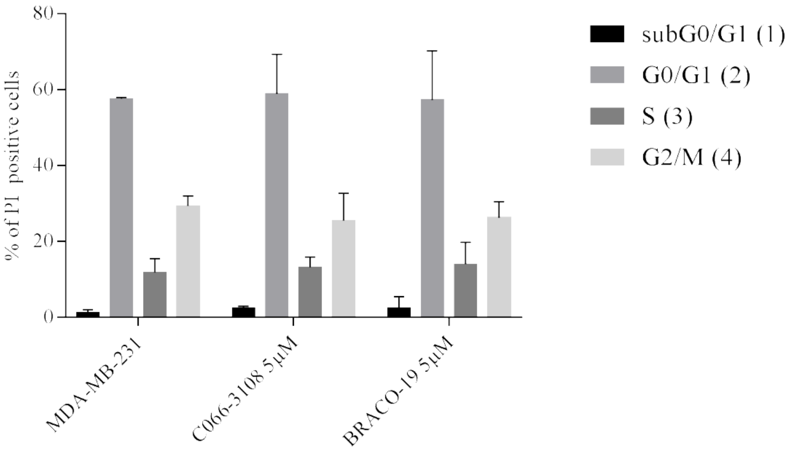

C

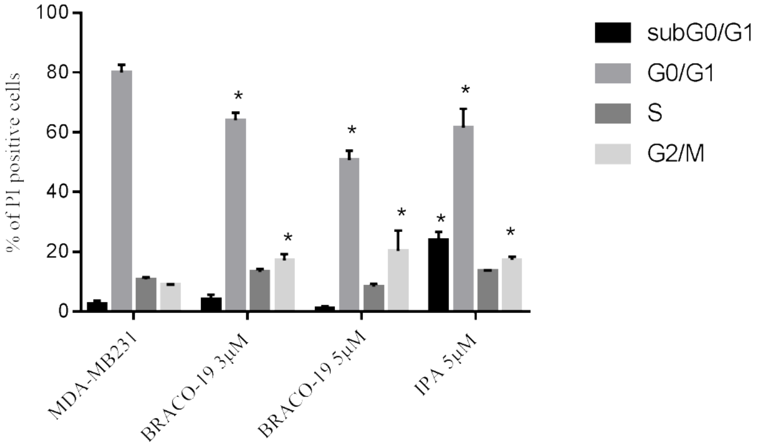

Figure S3. Cell cycle progression in MCF-7 and MDA-MB231. MCF-7 (**A**) and MDA-MB231 (**B**) cells were treated with G4 ligands at 5 μM for 3 days. Cells were stained with PI to evaluate cell cycle progression. The bars in the histograms represent the mean ± st.dev. of at least three independent experiments. No statistical significance was observed (calculated by GraphPad Prism 7 with two-way Anova using Sidak's multiple comparisons test). IPA was used as control compound at the concentration of 5 μM to validate the results of the cell cycle obtained at 6 days of culture. In (**C**), the effect of IPA is compared to the effect of BRACO-19 used at 3 and 5μM. The statistical significance was calculated by GraphPad Prism 7 with two-way Anova using Dunnett's multiple comparisons test (* $p < 0.05$).

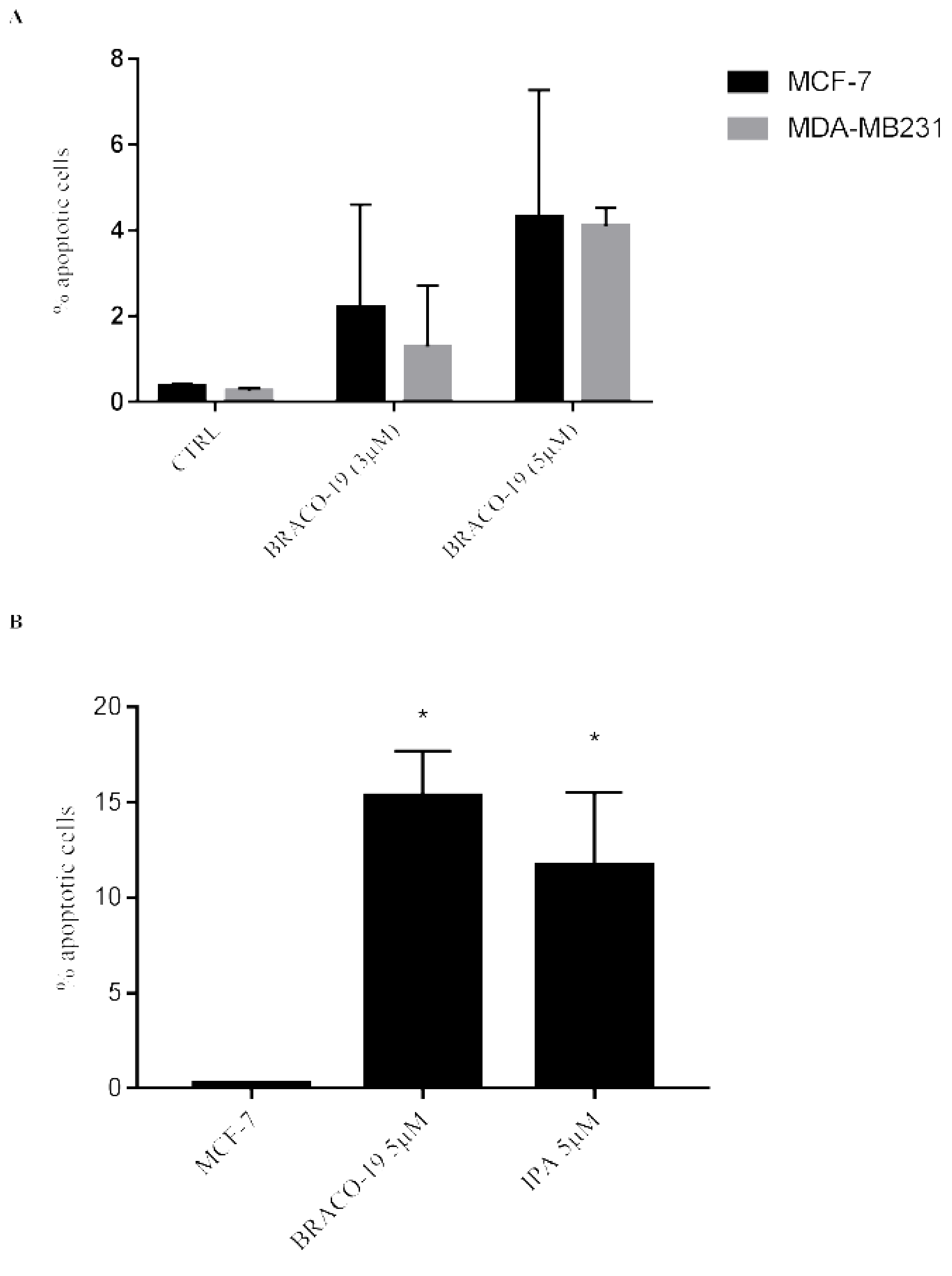

Figure S4. Apoptosis in MCF-7 and MDA-MB231. Apoptosis induction was evaluated by annexin V/PI staining at 72h of incubation with BRACO-19 at 3 and 5 µM (**A**). The percent of early apoptotic cells is reported the histogram that represents the mean ± st.dev. of three independent experiments. As control of apoptosis induction at 6 days of culture, IPA was used as control of apoptosis at the concentration of 5 µM in MCF-7 cells. In (**B**), the effect of BRACO-19 at 5 µM is compared to IPA at the same concentration. Early apoptotic cells are reported in the histogram that represents the mean of three independent experiments. The statistical significance was calculated by GraphPad Prism 7 with one-way Anova with Dunnett's multiple comparisons test (* $p < 0.05$).

A

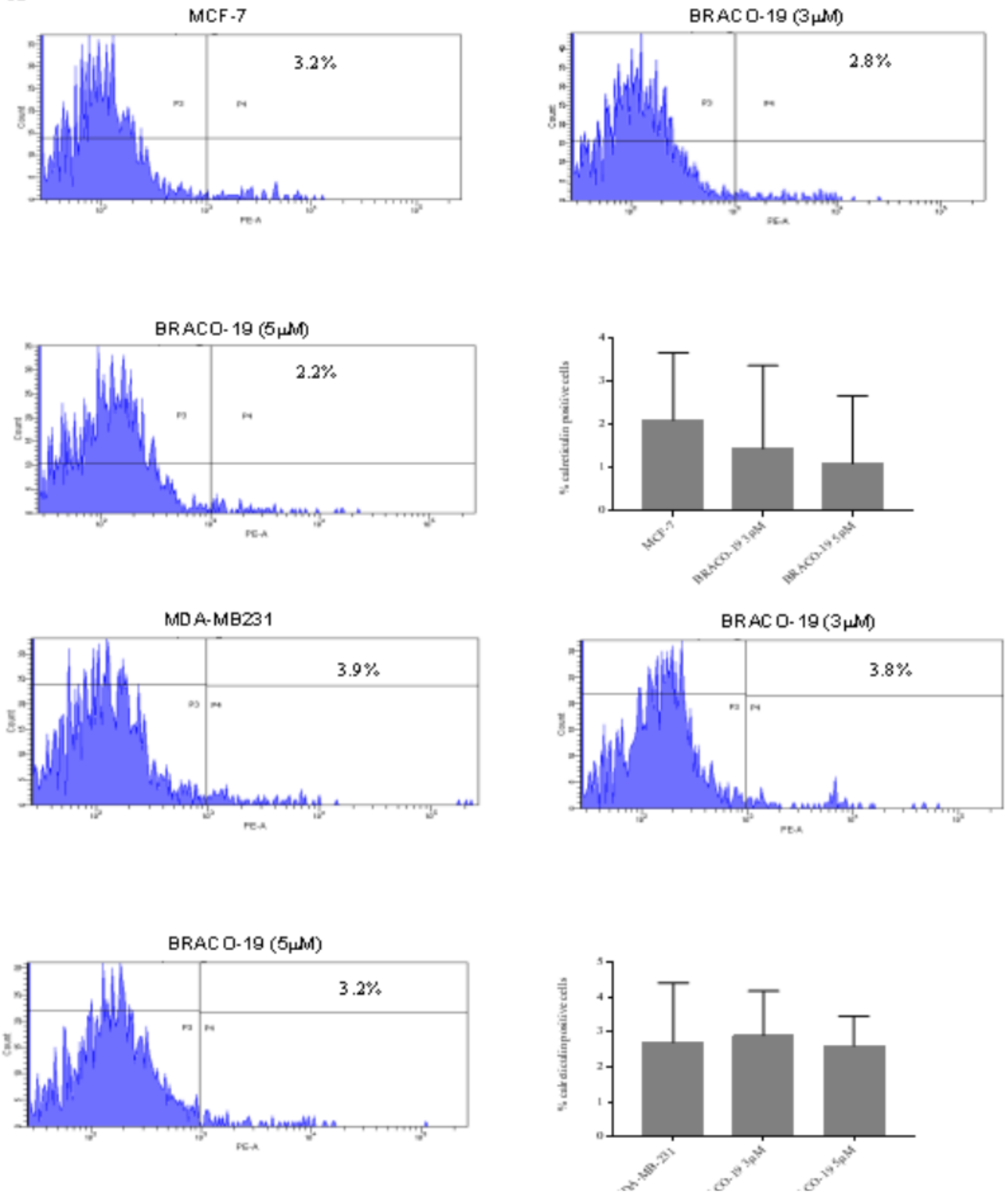
MCF-7
3.2%
BRACO-19 (3μM)
2.8%
BRACO-19 (5μM)
2.2%
% calreticulin positive cells
MCF-7
BRACO-19 3μM
BRACO-19 5μM
MDA-MB231
3.9%
BRACO-19 (3μM)
3.8%
BRACO-19 (5μM)
3.2%
% calreticulinpositivecells
PE-A
Count

B

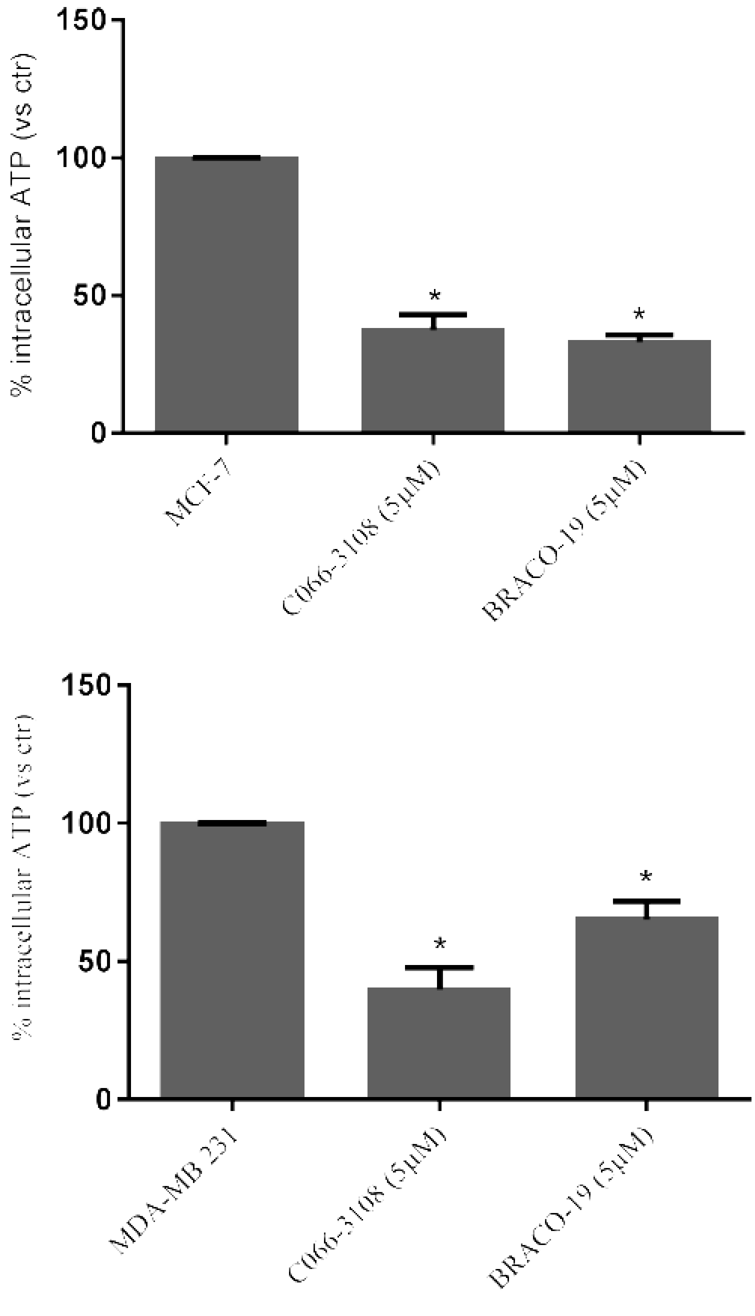
% intracellular ATP (vs ctr)
150
100
50
0
*
*
MCF-7
C066-3108 (5µM)
BRACO-19 (5µM)
% intracellular ATP (vs ctr)
150
100
50
0
*
*
MDA-MB 231
C066-3108 (5µM)
BRACO-19 (5µM)

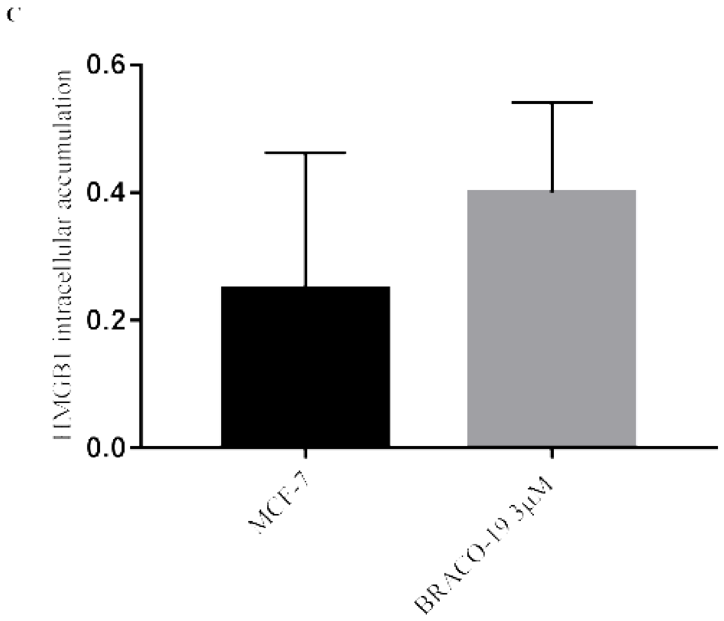

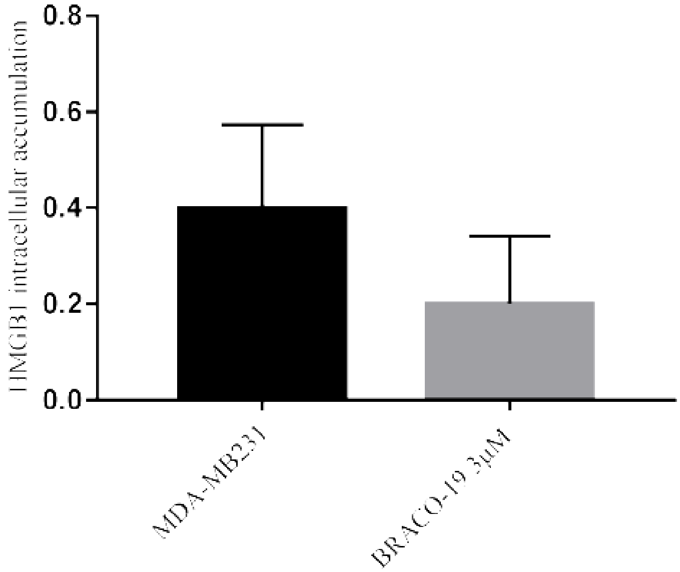

Figure S5. ICD hallmarks in MCF-7 and MDA-MB 231 cells treated with G4 ligands. (**A**) Calreticulin expression as surface cell marker was evaluated in MCF-7 and MDA-MB231 cells in the presence of BRACO-19 at 3 and 5 µM after 3 days of culture. Representative flow cytometry profiles of a single experiment are reported whereas the histograms represent the mean ± st.dev. of three independent experiments. (**B**) ATP intracellular release by MCF-7 and MDA-MB 231 treated with G4 ligands at 5 µM was determined as luminescence by microwell plate reader after 48 h of incubation, the histograms represent the mean ± st.dev. of three independent experiments. The statistical significance was calculated by GraphPad Prism 7 one-way Anova with Dunnett's multiple comparisons test (* $p < 0.05$). (**C**) HMGB1 intracellular accumulation is reported in the histograms for MCF-7 and MDA-MB231 cells at the concentration of 3 µM (histograms report the mean ± st.dev. of three independent experiments).
